# Diagnosis of rheumatic and autoimmune diseases dataset

**DOI:** 10.1016/j.dib.2025.111623

**Published:** 2025-05-07

**Authors:** Mohammed Fadhil Mahdi, Arezoo Jahani, Dhafar Hamed Abd

**Affiliations:** a Faculty of Electrical and Computer Engineering, Sahand University of Technology, Tabriz, Iran; b College of Computer Science and Information Technology University of Anbar Ramadi, Iraq

**Keywords:** Rheumatoid arthritis, Reactive arthritis, Ankylosing spondylitis, Sjögren syndrome, Systemic lupus erythematosus, Psoriatic arthritis

## Abstract

This study presents a primary dataset collected from one hospital and three laboratories in Iraq between 2019 and 2024. The dataset includes both the case and control groups, the case group comprising patients diagnosed with six common rheumatic and autoimmune diseases: rheumatoid arthritis, reactive arthritis, ankylosing spondylitis, Sjögren syndrome, systemic lupus erythematosus, and psoriatic arthritis. The dataset contains records of 12,085 patients with rheumatic and autoimmune diseases. Patient privacy is ensured through data anonymization. The dataset includes 14 features in seven classes, aiding the development of machine learning models for the early and accurate diagnosis of rheumatic and autoimmune diseases. This dataset is valuable for clinical decision support, remote healthcare, drug development, and medical diagnosis. It facilitates early diagnosis, supports explainable artificial intelligence models, advances precision medicine, enhances research, and reduces diagnostic costs and time.

Specifications Table

**Table:** 

Subject	Health and Medical Sciences, Computer Science
Specific subject area	Arthritis and Rheumatology, Machine Learning
Type of data	Raw data on rheumatic and autoimmune disease demonstrated in Excel file (dataset with label)
Data collection	The dataset for rheumatic and autoimmune diseases was collected from three independent laboratories (Al Hawraa Clinical, Al-Sibtain Medical, and Taqadum) and Saint Raphael Hospital. It includes detailed laboratory diagnoses obtained during routine diagnostics from 2019 to 2024. This dataset comprises 14 key features, including two demographic variables, age and sex, while the remaining features are derived from blood tests. Standardized methods were used for sample collection and analysis. Laboratory tests included anti-citrullinated peptide antibody (Anti-CCP ab), C-reactive protein (CRP), rheumatoid factor (RF), and complement proteins (C3 and C4), analyzed using Enzyme-Linked Immunosorbent Assay (ELISA) kits from Cusabio, China. Additional serological markers anti-Ro, anti-La, antinuclear antibodies (ANA), anti-double stranded DNA (anti-dsDNA), human leukocyte antigen B27 (HLA-B27), and anti-SM were tested using BioZik kits from the Netherlands. The dataset also includes the inflammatory marker erythrocyte sedimentation rate (ESR). All samples were reviewed by a rheumatology expert for classification. The dataset is labeled with seven categories: rheumatoid arthritis, reactive arthritis, ankylosing spondylitis, Sjögren’s syndrome, systemic lupus erythematosus, psoriatic arthritis, and a control (normal) group.
Data source location	**Hospital**: • **Saint Raphael**: Al-Karrada Street inside, Baghdad, Iraq. **Laboratory test:** • Al Hawraa Clinical, Al-Karrada, opposite the Dr. Abdul-Majeed Hospital, Baghdad, Iraq. • Al-Sibtain Medical LAB: Kasra, Al-Maghrib Street, Baghdad, Iraq. • Taqadum LAB: Cairo neighborhood street, Baghdad, Iraq.
Data accessibility	Repository name: Harvard Dataverse Data identification number: https://doi.org/10.7910/DVN/VM4OR3 Direct URL to data: https://dataverse.harvard.edu/dataset.xhtml?persistentId=doi%3A10.7910%2FDVN%2FVM4OR3&version=DRAFT
Related research article	None

## 1 Value of the Data

• Our dataset is the first specifically designed to diagnose rheumatic and autoimmune diseases early, making it suitable for machine learning and explainable artificial intelligence (XAI). Unlike existing datasets focusing on general autoimmune conditions. This dataset provides structured tabular data optimized for predictive modeling and feature analysis.
• The dataset has been collected from Iraqi patients, making it the first publicly available dataset representing Middle Eastern populations. It captures ethnic and genetic diversity, providing valuable insights for region-specific research.
• Researchers can use this dataset to identify subtitles for early detection of rheumatic and autoimmune diseases, improving the accuracy and timeliness of diagnoses for conditions such as rheumatoid arthritis, reactive arthritis, ankylosing spondylitis, etc.
• By including multiple rheumatic and autoimmune conditions, this dataset facilitates comparative studies, helping researchers identify shared biomarkers, risk factors, and treatment strategies across diseases.
• Supports the development of personalized treatment plans to improve outcomes and minimize side effects.
• Insights from this dataset can support cost-effectiveness studies, optimize resource allocation, and inform healthcare policy, ultimately improving access to early diagnostic tests and treatments.

## 2 Background

Rheumatology disease affects approximately 5%-10% of the population, and this rate is increasing [1]. This disease affects the joints, muscles, and connective tissues, causing inflammation, pain, swelling, and stiffness. Early diagnosis is crucial for proper treatment [2]. Generally, rheumatoid disease is divided into non-inflammatory and inflammatory [3]. Non-inflammatory diseases include ordinary degenerative diseases, such as osteoarthrosis, osteoarthropathy, and senile osteoporosis, which are related to genetic, environmental, and lifestyle. Inflammatory disease is categorized by immune system activation and is subdivided into two classes: infectious and non-infectious inflammatory [4]. Non-infectious inflammatory diseases include rheumatic and autoimmune diseases, such as rheumatoid arthritis, ankylosing spondylitis, and reactive arthritis, among others, whereas infectious rheumatic diseases are acute rheumatic fever [5]. Rheumatic and autoimmune diseases pose major challenges for diagnosis due to similarities in symptoms, different disease courses, and contamination of several cases due to the absence of particular biomarkers [6,7].

However, traditional diagnostic approaches often rely on the experience and interpretation of test results, which can lead to inaccuracies or delays in initiating treatment for some patients. Furthermore, so far no dataset has been specifically designed for the early diagnosis of autoimmune diseases. Most existing datasets focus on broad autoimmune disorders but lack precise diagnostic labels for conditions such as rheumatoid arthritis, Sjögren syndrome, and systemic lupus erythematosus.

This dataset is designed to support the early diagnosis of rheumatic and autoimmune diseases, ensuring optimal patient outcomes. Early detection of rheumatoid diseases allows a timely treatment with disease-modifying antirheumatic drugs (DMARDs), which slow disease progression and preserve joint function. This enables personalized treatment strategies to minimize disabilities and improve quality of life. However, early-stage symptoms often mimic other joint disorders, making diagnosis challenging even for expert rheumatologists. These complexities can lead to delayed or incorrect diagnoses, resulting in inappropriate treatment and, in some cases, irreversible damage.

## 3 Data Description

This dataset was specifically created to diagnose autoimmune and rheumatic diseases, containing real patient data collected from one hospital and three laboratories over different periods as shown in Table 1. The dataset is structured as a single-sheet Excel file that contains data from 12,085 patients.

**Table 1:** Number of instances from each hospital and laboratory tests with different duration.

No	Name of hospital and laboratory tests	Number of instances	Duration
1	Saint Raphael Hospital	3,953	2019 to 2024
2	Al Hawraa Clinical	828	2022 to 2024
3	Al-Sibtain Medical	3,879	2020 to 2024
4	Taqadum	3,425	2021 to 2024
**Total of instances**		**12,085**	

**Fig. 1:**
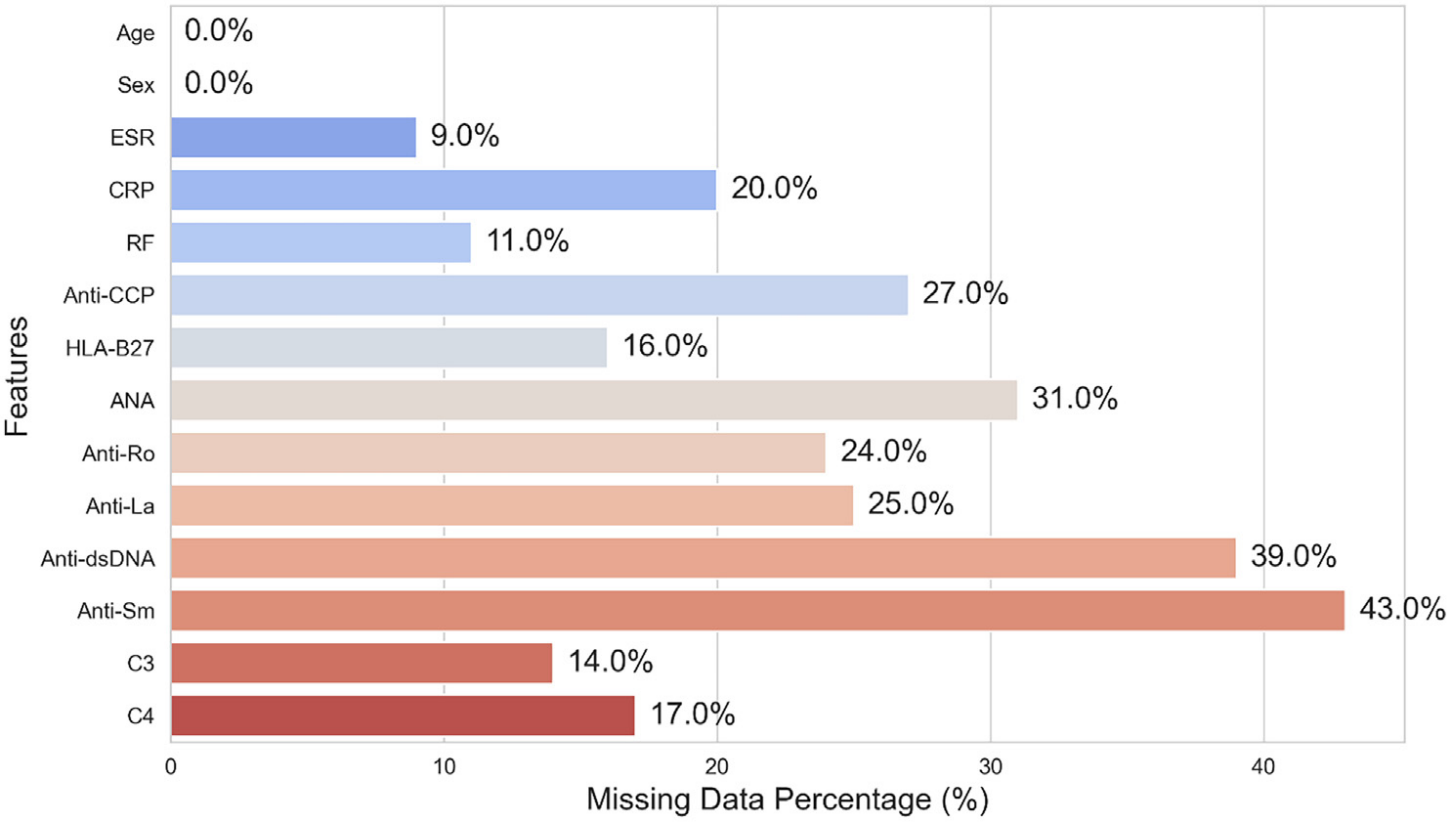
Missing data percentage for each class.

The clinical features revealed several key aspects, including data counts and missing data, as presented in Table 2 and Fig. 1. Age and sex features are complete, with no missing data. However, continuous features such as ESR, CRP, RF, and anti-CCP have missing data ranging from 9% to 27%. The binary features, including HLA-B27, ANA, Anti-Ro, and Anti-La, also contain missing data, with proportions between 16% and 43%. In particular, Anti-dsDNA and Anti-Sm exhibit the highest proportion of missing data at 43%. Meanwhile, C3 and C4 have moderate missing data, ranging from 14% to 17%. Additionally, there are no duplicate entries in the dataset.

**Table 2:** Missing data count for each feature.

No	Features	Missing data count
1	Age	0
2	Sex	0
3	ESR	1,088
4	CRP	2,417
5	RF	1,329
6	Anti-CCP	3,263
7	HLA-B27	1,934
8	ANA	3,746
9	Anti-Ro	2,900
10	Anti-La	3,021
11	Anti-dsDNA	4,713
12	Anti-Sm	5,197
13	C3	1,692
14	C4	2,054

**Fig. 2:**
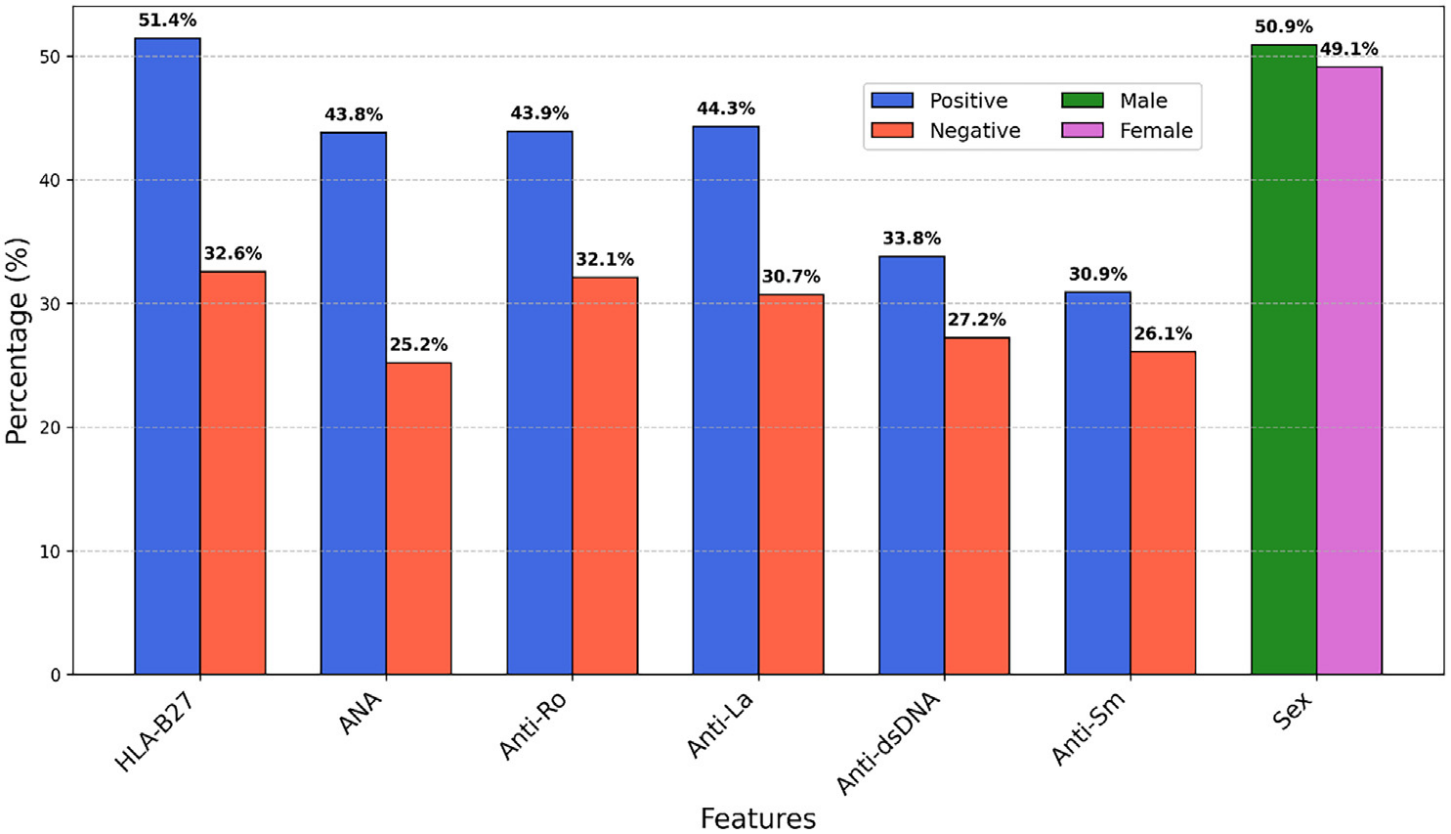
Percentage distribution of category features.

**Fig. 3:**
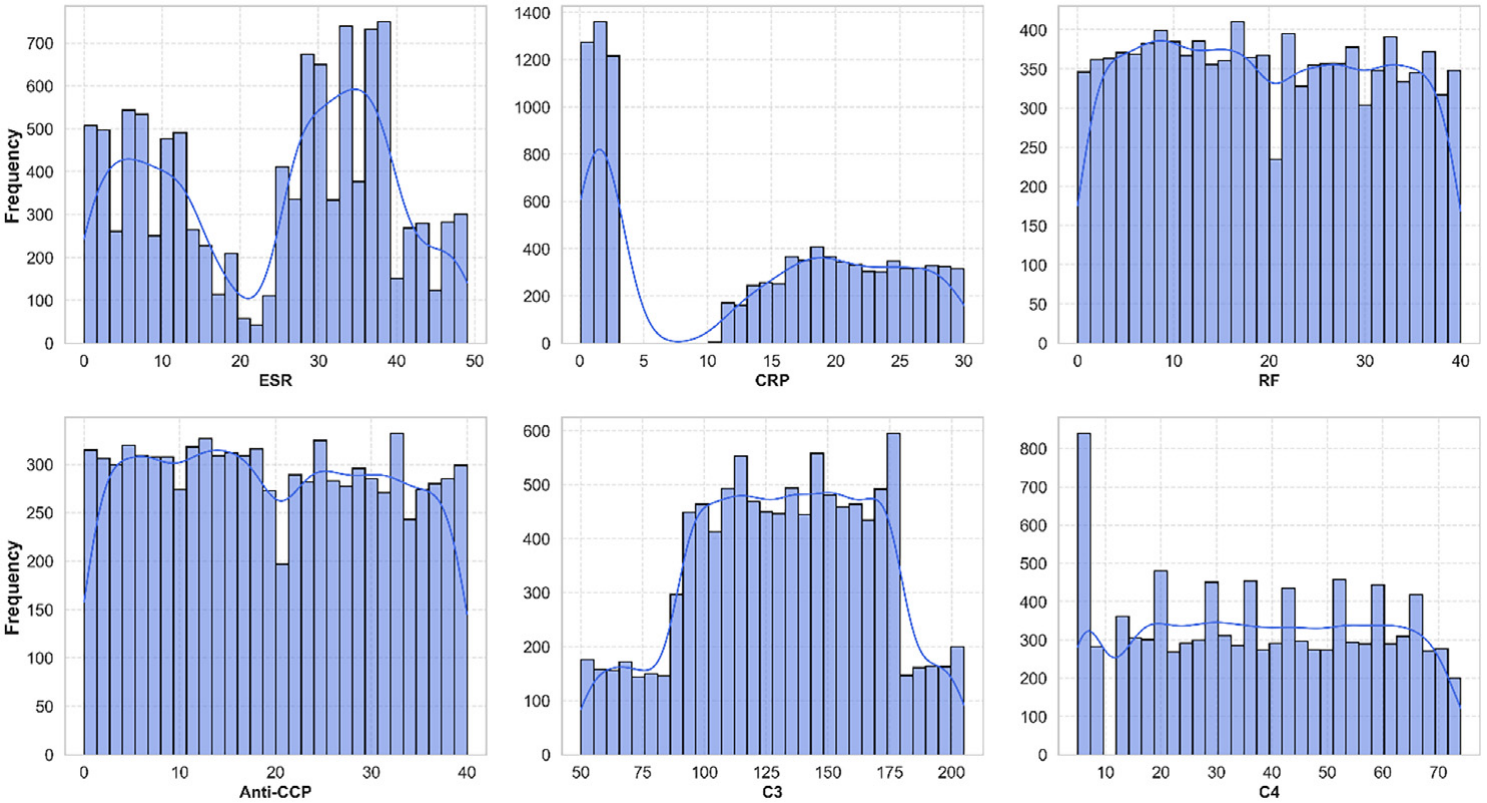
Distribution of numerical features.

The frequency distributions highlight the spread of data points, essential for identifying outliers, without considering missing data. Fig. 2 shows the distribution of categorical features (Anti-dsDNA, HLA-B27, ANA, Anti-Ro, Anti-La, Anti-Sm) and gender (Male/Female) in a dataset of rheumatic and autoimmune diseases. Fig. 3 illustrates the histogram distribution of six critical features (ESR, CRP, RF, Anti-CCP, C3, C4) used for diagnosing and differentiating rheumatic and autoimmune diseases, suggesting population heterogeneity and varying disease activity levels. Kernel Density Estimation (KDE) curves highlight the underlying distribution. No outliers were identified in these distributions.

Table 3 and Fig. 4 present the distribution of patients with various rheumatic and autoimmune diseases, along with the control (normal) population. Among these, rheumatoid arthritis is the most prevalent, with 2,848 cases, as it is more readily available in information systems. Ankylosing spondylitis and Sjögren syndrome have substantial representations, with 2,127 and 1,852 cases, respectively. Psoriatic arthritis accounts for 1,783 cases, while the control (normal) group includes 1,604 cases. The SLE class, with 1,355 cases, helps distinguish between healthy and diseased states, reducing false positives. The smallest class is reactive arthritis, with only 516 cases.

**Table 3:** Distribution of patients in classes of rheumatic and autoimmune diseases.

#	Classes for rheumatic and autoimmune diseases	Patient counts for each class
1	Rheumatoid arthritis	2,848
2	Reactive arthritis	516
3	Ankylosing spondylitis	2,127
4	Sjogren’s syndrome	1,852
5	systemic lupus erythematosus	1,355
6	Psoriatic arthritis	1,783
7	Control (normal)	1,604

**Fig. 4:**
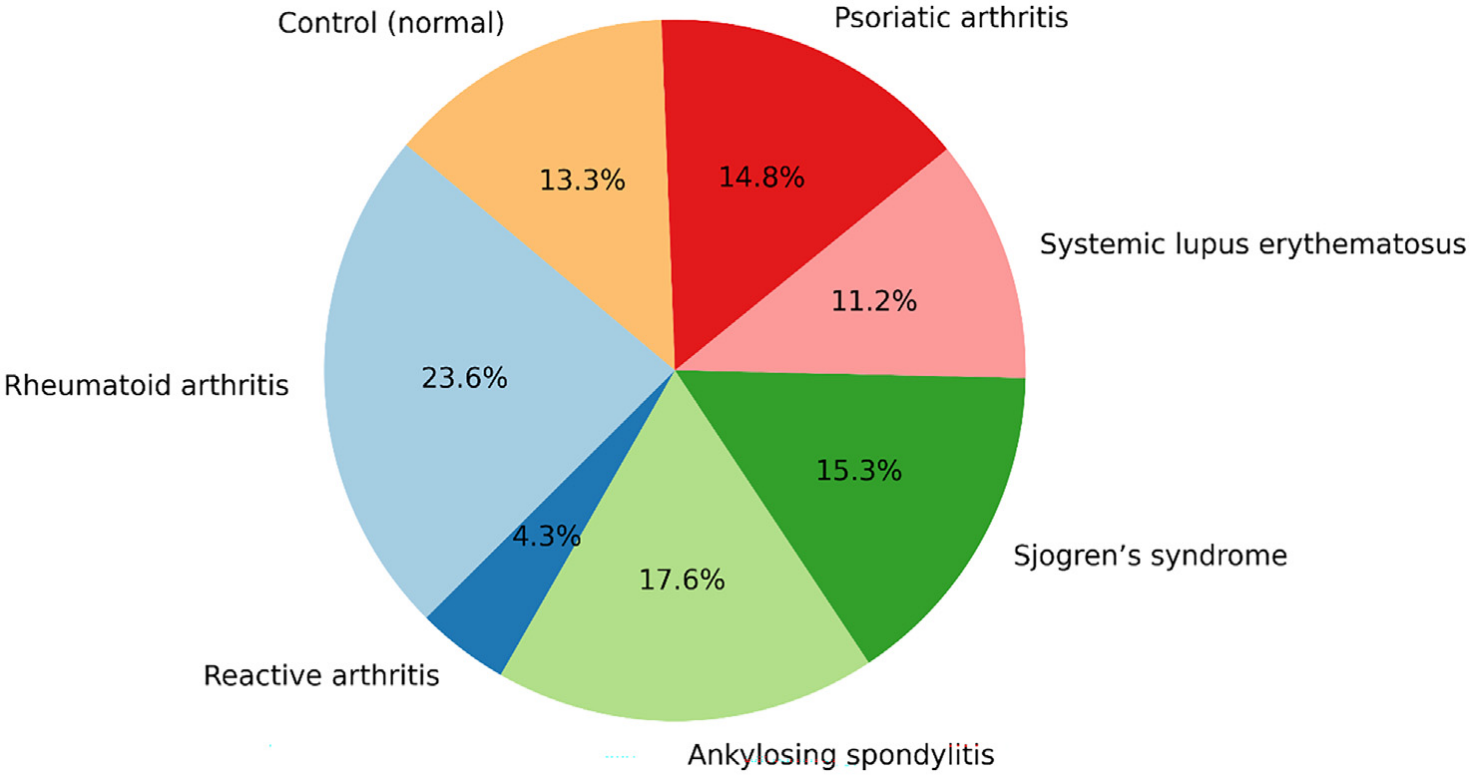
Percentage distribution of rheumatic and autoimmune disease classes.

## 4 Experimental Design, Materials and Methods

A total of 12,085 patients, including 1,604 classified as normal, were enrolled in this study. All participants experienced joint pain, which could be due to various conditions such as rheumatoid arthritis, reactive arthritis, ankylosing spondylitis, Sjögren syndrome, systemic lupus erythematosus, psoriatic arthritis, or normal. These individuals were referred from non-specialist clinics to receive comprehensive care and, if necessary, meet specific referral criteria for further evaluation.

**Fig. 5:**
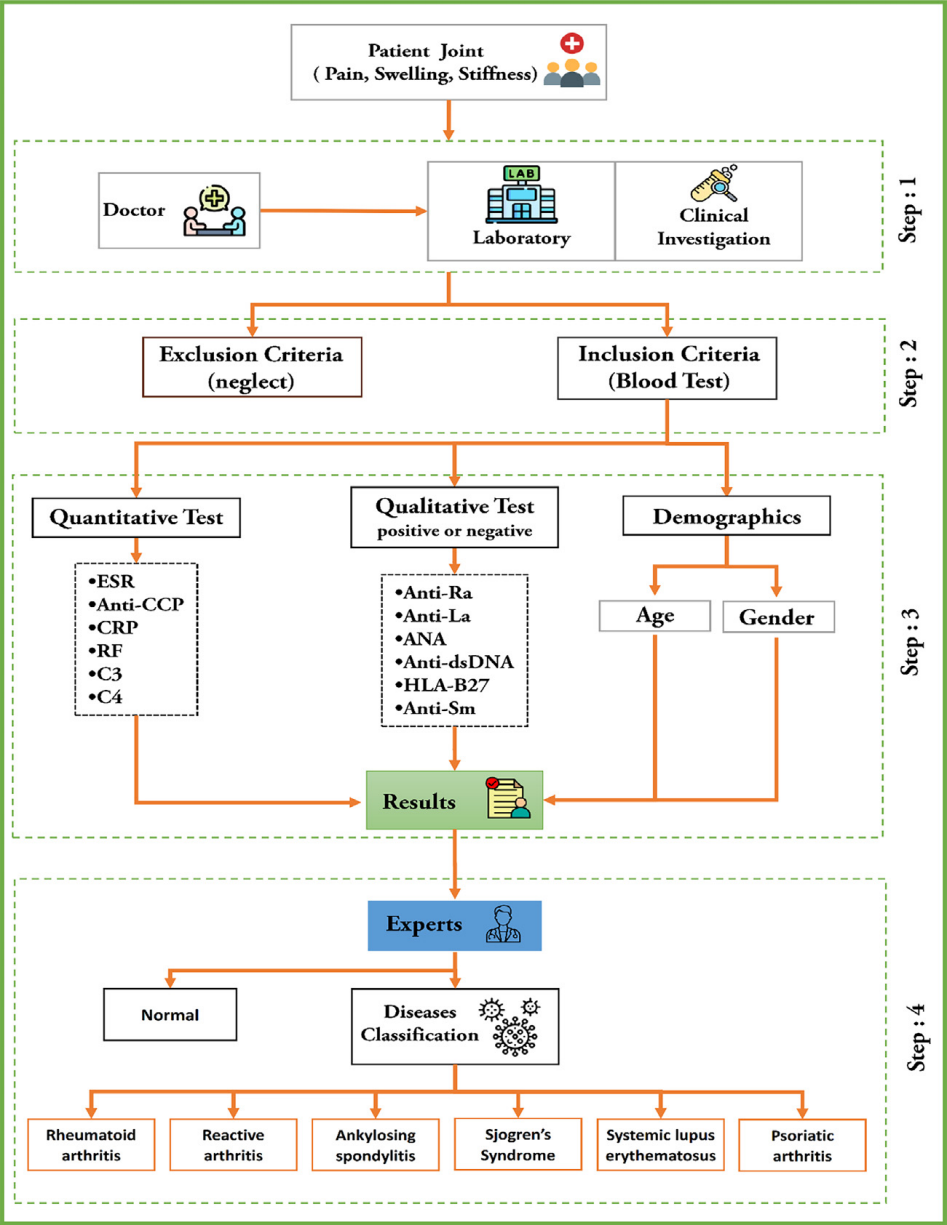
Steps in the process of collecting data for rheumatic and autoimmune diseases.

Biochemical parameters related to joint pain (including pain, swelling, and stiffness) were analyzed to determine their diagnostic significance. The results were collected and categorized into different disease classes according to the diagnosis, contributing to the development of a comprehensive patient dataset for rheumatoid and autoimmune diseases. Fig. 5 illustrates the four-step process for diagnosing rheumatic and autoimmune diseases through laboratory investigations.

The following four steps for collecting data for rheumatic and autoimmune diseases:

**Step 1:** Patients who had symptoms such as joint pain, stiffness, and/or swelling for more than 6 months were included in the study. Based on these symptoms, doctors have ordered laboratory tests to help diagnose the specific disease, as described in Table 4.

**Table 4:** Full set of features for autoimmune and rheumatic diseases dataset, which contains 14 features. The output is specified by the specialist expert.

No	Laboratory Test (Features)	Features Details
1	Age group	The patients are between 20 and 80 years old
2	Sex	The patients can be male or female.
3	ESR	For male patients, ESR values are in the normal range between 0 and 15 mm/hour (millimeter per hour), and for female patients, they are in the normal range between 0 and 20 mm/hour.
4	CRP	The CRP values for patients in the normal range are from 0.1 to 3.0 mg/L (milligrams per deciliter).
5	RF	RF values for patients in the normal range are from 0.1 to 3.0 IU/ml (international unit per liter)
6	Anti-CCP	values for patients in the normal range are from 0.0 to 20.0 U/mL (unit per milliliter)
7	HLA-B27	Binary values indicate either a positive or negative result. A negative result means that the antibody was not detected, while a positive result confirms its presence in the body.
8	ANA	Binary values indicate either a positive or negative result. A negative result means that the antibody was not detected, while a positive result confirms its presence in the body.
9	Anti-Ro	Binary values indicate either a positive or negative result. A negative result means that the antibody was not detected, while a positive result confirms its presence in the body.
10	Anti-La	Binary values indicate either a positive or negative result. A negative result means that the antibody was not detected, while a positive result confirms its presence in the body.
11	Anti-dsDNA	Binary values indicate either a positive or negative result. A negative result means that the antibody was not detected, while a positive result confirms its presence in the body.
12	Anti-Sm	Binary values indicate either a positive or negative result. A negative result means that the antibody was not detected, while a positive result confirms its presence in the body.
13	C3	For male patients, normal C3 values range between 90 and 180 mg/dL, while for female patients, they range between 88 and 206 mg/dL.
14	C4	For male patients, normal C4 values range between 12 and 72 mg/dL, while for female patients, normal C4 values range between 13 and 75 mg/dL
15	Disease	Output
		Rheumatoid arthritis, reactive arthritis, ankylosing spondylitis, Sjogren syndrome, systemic lupus erythematosus, or psoriatic arthritis.

**Step 2:** Patients were selected on specific inclusion and exclusion criteria. The inclusion criteria required participants to experience persistent small joint pain in the hands and feet (both symmetrical and asymmetrical) for more than 6 months. Exclusion criteria included patients with elevated uric acid levels (>7.0 mg/dL), indicative of gout disease, as well as those diagnosed with COVID-19, other acute influenza conditions, or malignancies.

**Step 3:** Clinical investigations were carried out using standardized laboratory methods for collecting and analyzing quantitative and qualitative diagnostic measures. Quantitative tests included anti-CCP, CRP, RF, C3, and C4, analyzed using Cusabio, China, ELISA kits. Qualitative diagnostic tests (positive or negative) included anti-Ro, anti-La, ANA, anti-dsDNA, HLA-B27, and anti-SM, which were assessed using serological kits from BioZik, The Netherlands. In addition, the inflammatory marker ESR was measured.

**Step 4:** A specialist in Rheumatology and Medical Rehabilitation with 10 years of expertise in the field reviewed the laboratory test results and classified the patients based on the type of rheumatic and autoimmune disease. Based on laboratory findings, patients were diagnosed with one of the following rheumatic and autoimmune diseases: rheumatoid arthritis, reactive arthritis, ankylosing spondylitis, Sjögren’s syndrome, systemic lupus erythematosus, psoriatic arthritis or categorized as normal.

Table 4 provides valuable information through 14 key features, which consist of demographic information and laboratory test results, that help diagnose and assess. Features details have the normal reference ranges for biochemical and immunological markers used in assessing autoimmune and rheumatic diseases. It includes numerical values that indicate normal range, as well as binary markers that are either positive or negative. A negative result is generally considered normal, while a positive result suggests a potential autoimmune response requiring further clinical evaluation. Additionally, Row 15 in Table 4 categorizes patients based on their laboratory results, linking specific biomarker variations to diagnosed conditions such as rheumatoid arthritis, ankylosing spondylitis, Sjögren’s syndrome, systemic lupus erythematosus, psoriatic arthritis, and reactive arthritis. This classification helps in distinguishing between control (normal) individuals and those affected by autoimmune and rheumatic diseases.

## Limitations

Despite the uniqueness of our dataset, we identified three limitations that can impact the performance of the training model. First, the dataset has an imbalanced class distribution, which can bias the model toward the majority class and reduce its generalization to minority classes. Techniques such as the Synthetic Minority Oversampling Technique (SMOTE) can be applied to address this issue. Second, missing values in many features can affect the accuracy and reliability. A solution is to implement imputation methods, such as mean, mode, or median, for numerical and categorical data, or apply predictive imputation techniques for more robust handling. Finally, while the dataset includes 12,085 instances, collecting additional data could improve the model’s ability to learn complex patterns and improve generalization. This can be addressed by gathering more data or employing data augmentation techniques.

## Data Availability

DataverseRheumatic and autoimmune diseases dataset (Original data).
